# Disruption of an *M. tuberculosis* Membrane Protein Causes a Magnesium-dependent Cell Division Defect and Failure to Persist in Mice

**DOI:** 10.1371/journal.ppat.1004645

**Published:** 2015-02-06

**Authors:** Nichole Goodsmith, Xinzheng V. Guo, Omar H. Vandal, Julien Vaubourgeix, Ruojun Wang, Hélène Botella, Shuang Song, Kamlesh Bhatt, Amir Liba, Padmini Salgame, Dirk Schnappinger, Sabine Ehrt

**Affiliations:** 1 Department of Microbiology and Immunology, Weill Cornell Medical College, New York, New York, United States of America; 2 Department of Medicine, Center for Emerging Pathogens, Rutgers, The State University of New Jersey, New Jersey Medical School, Newark, New Jersey, United States of America; 3 Agilent Technologies, Wilmington, Delaware, United States of America; McGill University, CANADA

## Abstract

The identification of *Mycobacterium tuberculosis* genes necessary for persistence *in vivo* provides insight into bacterial biology as well as host defense strategies. We show that disruption of *M. tuberculosis* membrane protein PerM (Rv0955) resulted in an IFN-γ-dependent persistence defect in chronic mouse infection despite the mutant’s near normal growth during acute infection. The *perM* mutant required increased magnesium for replication and survival; incubation in low magnesium media resulted in cell elongation and lysis. Transcriptome analysis of the *perM* mutant grown in reduced magnesium revealed upregulation of cell division and cell wall biosynthesis genes, and live cell imaging showed PerM accumulation at the division septa in *M. smegmatis*. The mutant was acutely sensitive to β-lactam antibiotics, including specific inhibitors of cell division-associated peptidoglycan transpeptidase FtsI. Together, these data implicate PerM as a novel player in mycobacterial cell division and pathogenesis, and are consistent with the hypothesis that immune activation deprives *M. tuberculosis* of magnesium.

## Author Summary

The success of *Mycobacterium tuberculosis* (Mtb) as a human pathogen is due to ability to persist in chronic infection, despite a robust adaptive immune response by the host. The mechanisms by which Mtb achieves this are, however, poorly understood. Here we show that a novel integral membrane protein, Rv0955/PerM, is essential for Mtb persistence during chronic mouse infection. The *perM* mutant required increased magnesium compared to wild type Mtb for replication and survival in culture and elongated in media with reduced magnesium concentration. Transcriptomic, electron microscopy and live cell imaging approaches provided evidence that PerM is involved in cell division. The survival defects of the *perM* mutant in reduced magnesium and during chronic mouse infection are consistent with the hypothesis that magnesium deprivation constitutes an IFN-γ dependent host defense strategy. This work also has potential clinical implications, as disruption of PerM renders Mtb susceptible to β-lactam antibiotics, which are commonly used to treat non-mycobacterial infections.

## Introduction

With an estimated one-third of the world’s population latently infected with *Mycobacterium tuberculosis* (Mtb), the question remains: how is this pathogen able to persist *in vivo*? In the mouse model, Mtb infection is characterized by an acute phase of logarithmic bacterial growth lasting approximately three weeks, followed by a plateau in bacterial burden, persisting as a chronic infection. The transition from acute to chronic infection—from logarithmic bacterial growth to stable bacterial counts—results from the onset of the adaptive immune response and activation of host macrophages by CD4+ T cell-derived IFN-γ [1,2]. IFN-γ enhances the antimicrobial capacity of macrophages by numerous mechanisms including promotion of phagosome maturation and acidification via induction of the GTPase Irgm1 and production of reactive nitrogen and oxygen species mediated by nitric oxide synthase and phagocyte oxidase [3–6]. However, IFN-γ induces hundreds of genes in macrophages [7] and the array of environmental modifications occurring within these macrophages and leading to control of Mtb growth is not entirely understood.

Mtb persistence mutants (*per* mutants) are a unique class of strains that are competent for replication during acute infection, but attenuated during chronic infection [8]. Several previously identified *per* mutants provide information about the processes required for survival in the activated macrophage following the onset of adaptive immunity. For example, a *per* phenotype was observed for an Mtb mutant lacking isocitrate lyase-1, an enzyme involved in the glyoxylate shunt and methylcitrate cycle, as well as a mutant lacking the cholesterol transporter Mce4, indicating that cholesterol and fatty acids are carbon sources required by Mtb to survive during chronic infection [9,10].

Macrophage activation promotes phagosomal maturation and intraphagosomal acidification [6,11,12]. In a screen for Mtb transposon mutants hypersusceptible to acid stress, we previously identified 21 genes whose interruption lead to reduced viability in low pH [13]. The majority of these genes are annotated to have functions related to cell wall processes. These included two independent transposon mutants of the previously uncharacterized Mtb gene *rv0955*, a 1,368 base pair open reading frame, which is annotated to encode an integral membrane protein with a predicted topology of ten transmembrane helices (S1 Fig.) [14–16]. *Rv0955* is highly conserved among mycobacteria and actinobacteria, but has no known homologues in other species, and no conserved sequence motifs to predict its function. It is included among the 219 mycobacterial “core” genes noteworthy for their conservation among mycobacterial species, including Mtb and *M*. *leprae* [17]. These core genes lack homologues in other bacteria, suggesting that their function may be unique to mycobacteria, and making them potential targets for mycobacteria-specific drugs.

Here, we investigated the function of the previously uncharacterized Mtb Rv0955 protein. Disruption of *rv0955* resulted in a striking persistence defect in chronic mouse infection with a 300-fold decline in bacterial burden in the lungs. We therefore named this gene *perM*, encoding a persistence-associated integral membrane protein. As Vandal et al. noted, the acid susceptibility of the *perM* mutant—similar to many of the mutants identified in the screen—was detergent-dependent, observed only when the bacteria were exposed to a combination of low pH and Tween-80 detergent [13]. We thus sought to investigate mechanisms beyond protection from acid, which might account for the strong attenuation of the mutant *in vivo*.

We found that the *perM* mutant required increased magnesium (Mg^2+^) compared to wild type (wt) Mtb for replication and survival in culture. Mg^2+^ is among the most abundant divalent cations in both prokaryotic and eukaryotic cells, and is essential for bacterial growth. In bacteria, Mg^2+^ serves a wide range of roles: it functions as a cofactor with ATP in numerous enzymatic reactions, enables the formation of tRNA and ribosomal tertiary structure, and regulates stability of the cell wall and membrane [18–20]. Mg^2+^ also impacts virulence in *Salmonella enterica* by regulating the PhoP/PhoQ two-component system [21].

In Mtb, two Mg^2+^-dependent mutants have been identified: Mtb∆*phoP* and Mtb∆*mgtC* [22,23]. PhoP shows high similarity to the PhoP response regulator of *Salmonella enterica* and is required in Mtb for the synthesis of several complex cell wall lipids as well as replication in macrophages and mice [22,24,25]. MgtC is required for virulence of both Mtb and *Salmonella enterica* and inhibits the bacterial F_1_F_0_ ATP synthase to maintain physiological ATP levels and intrabacterial pH [23,26].

Mg^2+^ restriction remains a plausible but unconfirmed antimycobacterial mechanism employed by the host. In media with low Mg^2+^ concentrations, the *perM* mutant elongated and upregulated expression of cell division and cell wall biosynthesis genes. Furthermore, Mtb PerM accumulated at the putative division septa in the closely related *M*. *smegmatis*. Disruption of *perM* resulted in pronounced hypersusceptibility to beta-lactam antibiotics, including cephalexin and piperacillin, which are specific inhibitors of the cell division-associated peptidoglycan synthesis protein FtsI. This work characterizes a novel mycobacterial protein necessary for persistence *in vivo* and implicated in cell division, and is consistent with the hypothesis that Mtb has reduced access to Mg^2+^ during chronic infection.

## Results

### PerM is required for Mtb persistence *in vivo*

**Fig 1:**
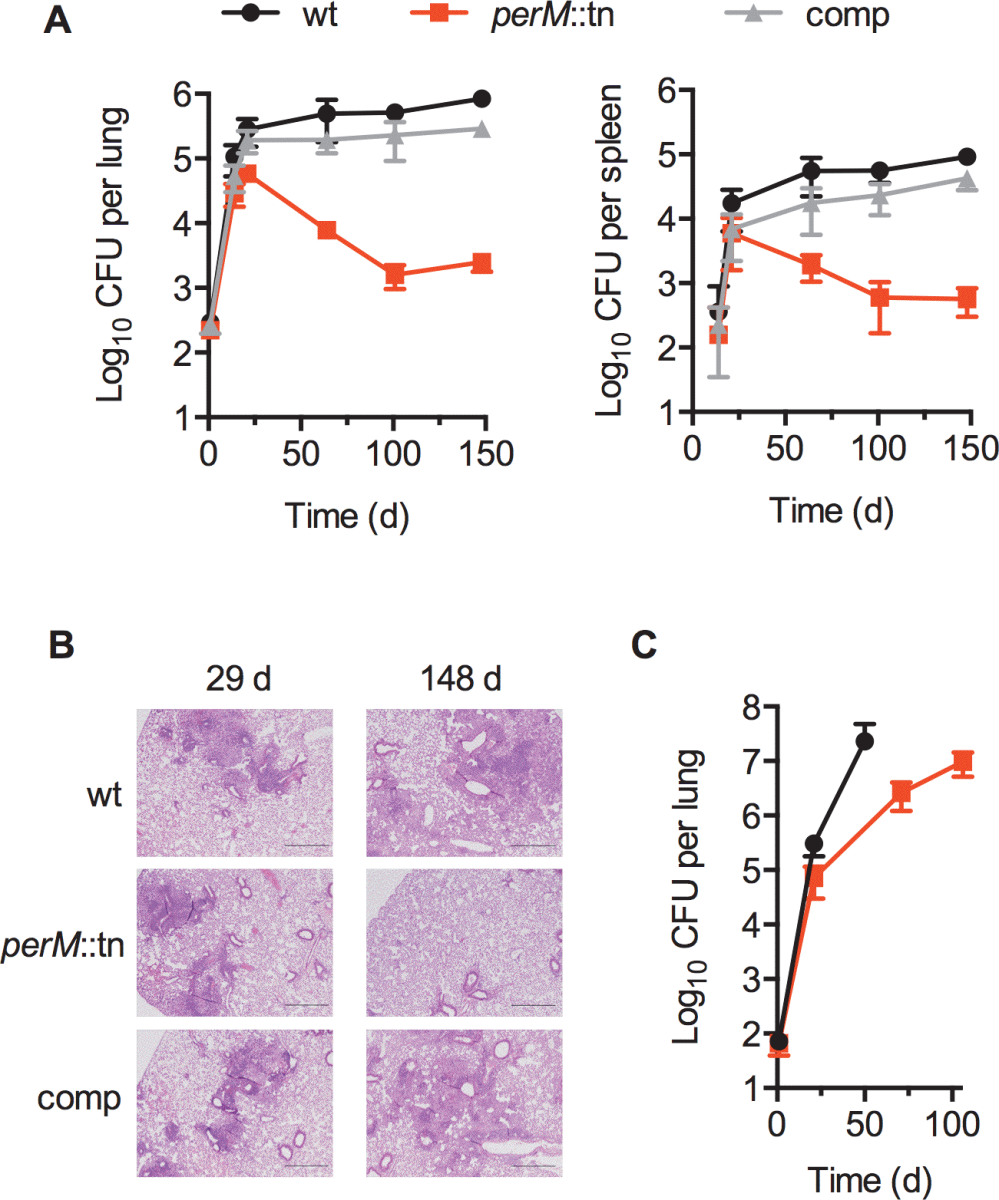
PerM is necessary for Mtb persistence *in vivo* in an IFN-γ-dependent manner. (**A**) Colony-forming units (CFU) in lungs (left) and spleens (right) from C57BL/6 mice infected by aerosol with H37Rv (wt), the *perM* transposon mutant (*perM*::tn), or the genetically complemented mutant (comp). Data are means ± SD of 4 mice and representative of three independent experiments. (**B**) Hematoxylin and eosin-stained lung sections of infected mice showing progression of lesions. (**C**) CFU in lungs of IFN-γ^-/-^ mice infected by aerosol with wt or *perM*::tn. Data are means ± SD of 4 mice.

*PerM* was previously identified in a screen for Mtb genes required for acid resistance [13]. To examine the role of PerM *in vivo*, we monitored replication and survival of a *perM* transposon mutant, *perM*::tn, in wild type mice. *PerM*::tn established infection and replicated during the acute phase, with only a 5-fold reduction in peak bacterial burden, measured by colony forming units (CFU), compared to wt (P = 0.032) at 21 days (Fig. 1A). However, *perM*::tn exhibited a severe persistence defect in chronic infection, with a 300-fold reduction in CFU in the lungs at fourteen weeks post-infection.

In agreement with these growth patterns, histological analysis revealed markedly fewer and smaller lesions in *perM*::tn-infected lung tissue compared to wt Mtb-infected mice, a difference observed at the 148 day post-infection time point, but not at the end of acute infection (Fig. 1B). Genetic complementation of the *perM* mutant with a wild-type copy of the gene expressed chromosomally under control of the hsp60 promoter restored persistence and increased granulomatous inflammation, indicating that attenuation *in vivo* was due to disruption of *perM*.

The adaptive immune response to Mtb is characterized by IFN-γ mediated activation of host macrophages. To examine whether death of *perM*::tn *in vivo* was dependent on host IFN-γ, we infected IFN-γ knockout mice, which are unable to control replication of wt Mtb [1,2]. *PerM*::tn replicated in IFN-γ knockout mice, but at a slower rate than wt Mtb (Fig. 1C). IFN-γ knockout mice infected with wt Mtb had to be sacrificed at day 50, because they were moribund, in contrast to IFN-γ knockout mice infected with *perM*::tn, which remained healthy through the end of the experiment (day 106). These results indicate that killing of *perM*::tn in wt mice requires host IFN-γ, while the mutant also exhibits an IFN-γ-independent replication defect.

### *PerM*::tn stimulates a hyperinflammatory cytokine response in infected macrophages *ex vivo*

**Fig 2:**
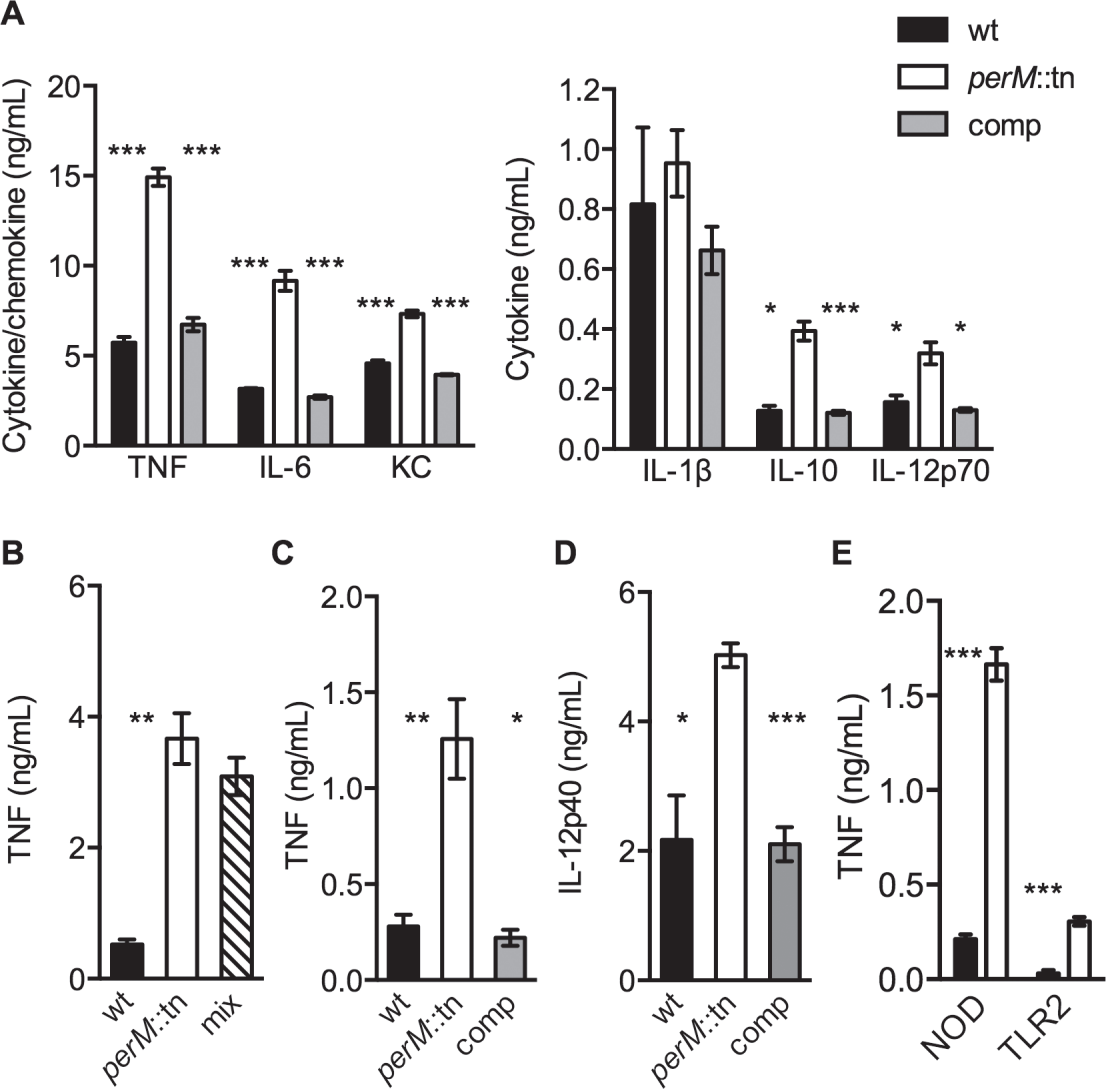
*PerM*::tn induces a hyperinflammatory cytokine response in mouse macrophages. (**A**-**D**) Cytokine content in cell culture supernatant of wt BMDM exposed for 24 hours to: (**A**) live Mtb at multiplicity of infection (MOI) 10; (**B**) live wt, *perM*::tn, or an equal mix of wt and *perM*::tn at total MOI of 10; (**C**) formalin-killed Mtb at MOI 20; (**D**) sterile, Mtb-conditioned media from 8 day old cultures. Cytokines/chemokines were not detectable in uninfected BMDM. (**E**) TNF production of a NOD1/NOD2^-/-^ cell line derived from B6 mice (NOD) or TLR2^-/-^ BMDM (TLR2) infected with wt or *perM*::tn at MOI 20. Protein concentration measured by ELISA. Data are means ± SD of three biological replicates. * P< 0.05, ** P< 0.01, *** P< 0.005.

Since later-occurring persistence defects like that of *perM*::tn often depend on the adaptive immune response of the host, we hypothesized that PerM might cause a more robust immune response than wt Mtb. To examine this possibility, we infected bone marrow derived mouse macrophages with equal numbers of wt, *perM*::tn and complemented mutant and measured cytokine concentrations in macrophage culture supernatants 24 hours later. Supernatants of macrophages infected with *perM*::tn contained elevated levels of proinflammatory cytokines, including TNF-α, IL-6, IL-12 p70, the anti-inflammatory cytokine IL-10, and the chemokine KC (Fig. 2A).

To assess the immune response to *perM*::tn during mouse infection we measured cytokine transcripts in mouse lungs by quantitative real-time polymerase chain reaction (qRTPCR), focusing on pro-inflammatory cytokines required to attenuate Mtb growth *in vivo* [1,27]. We did not observe significant differences in IFN-γ or TNF-α mRNA levels at 2 weeks post-infection, when bacterial titers of *perM*::tn were 3-fold lower than those of wt (S2A–S2B Fig.). In an independent experiment, IL-12p40 protein levels in lung homogenates from mice infected with wt or *perM*::tn were similar at 1 or 2 weeks post-infection and increased in wt compared to mutant infected lungs or 3 weeks post-infection (S2C–S2D Fig.). Differences in CFU confounded interpretation of these data, as bacterial counts of *perM*::tn were 3-, 5- and 6-fold reduced compared to wt at weeks 1, 2 and 3, respectively; however, these results suggested that attenuation of the mutant *in vivo* was not exclusively due to a more robust immune response that preceded in *perM*::tn-infected mice.

We sought to better understand the properties of *perM*::tn leading to the increased innate immune response by macrophages infected *ex vivo*. In a mixed-strain Mtb infection of macrophages, TNF-α production was similar to that induced by infection with *perM*::tn alone at the same total multiplicity of infection (MOI) (Fig. 2B). The dominance of the mutant suggested that the difference in response to these strains was due to an immunostimulatory effect of the mutant, as opposed to a suppressive effect of intact PerM protein produced by wt Mtb. The stimulatory effect of *perM*::tn was reproduced by exposure of macrophages to formalin-killed Mtb (Fig. 2C) and to cell-free Mtb-conditioned culture media (Fig. 2D), indicating that the stimulatory component(s) were shed or secreted by live *perM*::tn, but did not require viable bacteria for production or release during macrophage infection. In the absence of NOD and TLR2 signaling, *perM*::tn still elicited higher levels of TNF-α than wt (Fig. 2E). NOD and TLR2 are required for the macrophage response to bacterial peptidoglycan and triacylated lipoproteins, respectively, suggesting that the hyperinflammatory phenotype of *perM*::*tn* is not tied specifically to one of these cell wall components. TNF-α production was, however, significantly lower in cultures from knockout macrophages compared to wt macrophages, indicating that these receptors are important for TNF-α production following infection with both strains. Together, these data suggest that a combination of cellular components, both released into the medium during growth and expressed on the surface of killed *perM*::tn, function to stimulate increased inflammatory signaling in macrophages.

### PerM is necessary for growth and survival in low magnesium

**Fig 3:**
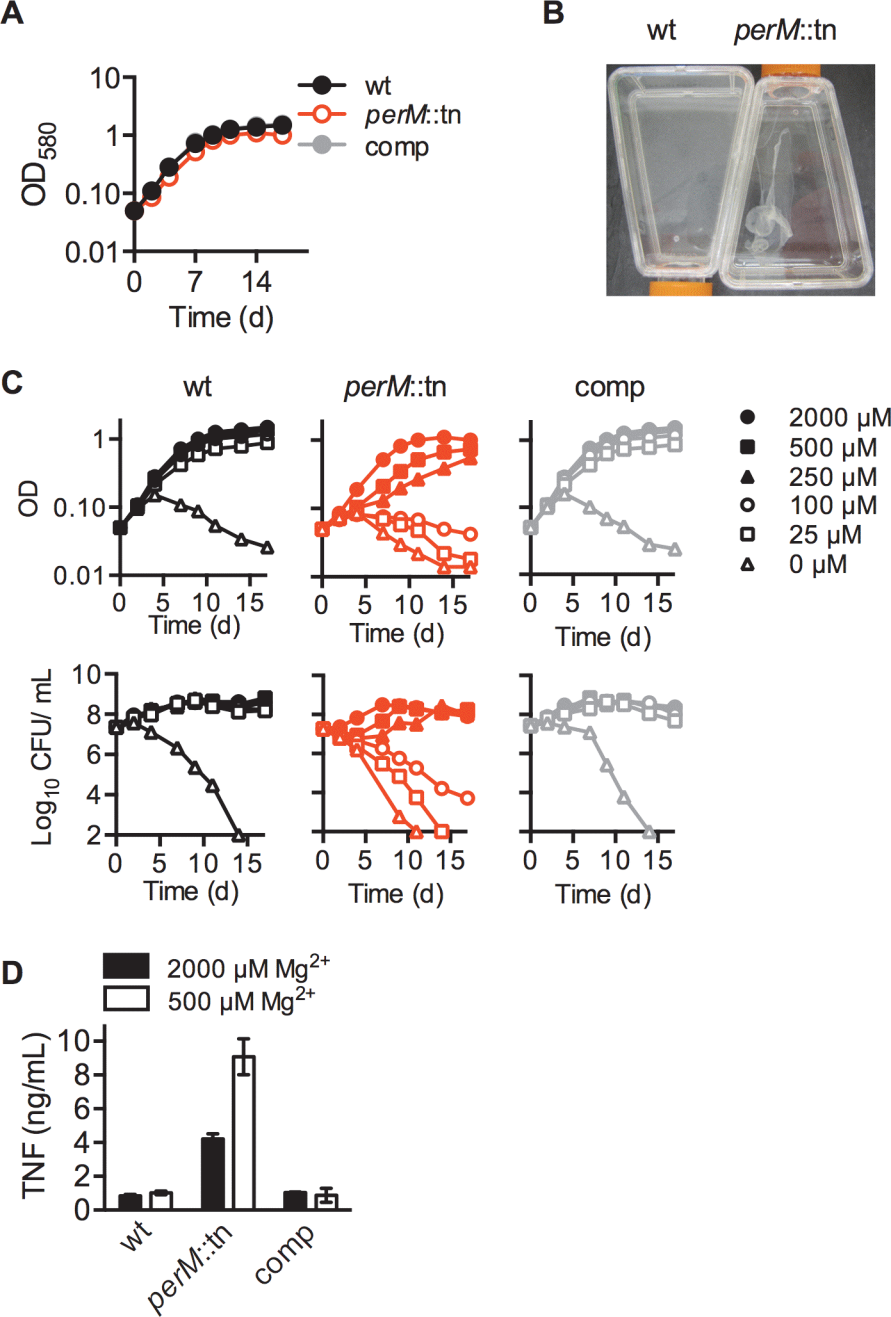
PerM is required for replication and survival in reduced magnesium. (**A**) Growth measured by optical density (OD_580_) of strains grown in Sauton’s minimal media with 0.05% Tween-80. (**B**) Macroscopic appearance of liquid cultures. Cultures were grown standing in Sauton’s media, then agitated carefully to visualize the disrupted film in *perM*::tn cultures. (**C**) Replication, measured by OD (above), and survival, measured by CFU (below), of Mtb grown at the indicated concentrations of Mg^2+^ added to nominally Mg^2+^-free Sauton’s minimal media. (**D**) TNF concentration in cell culture supernatant of bone marrow-derived mouse macrophages exposed for 24 hours to live Mtb (MOI 10), following 6 days of Mtb culture in 500 or 2000 μM Mg^2+^. Data are means ± SD of three replicates and representative of two independent experiments.

In liquid media, *perM*::tn replicated at a near-normal rate (Fig. 3A), but formed a loose aggregate during growth (Fig. 3B). Unlike previously described mycobacterial biofilms [28], these aggregates formed on the bottom of standing cultures, rather than at the liquid-air interface, and could be readily dispersed by shaking or pipetting. These aggregates suggested a perturbation of the *perM*::tn cell envelope. Since extracellular magnesium (Mg^2+^) has been shown to overcome phenotypes of mutants with cell envelope defects [22], we asked whether reduction of Mg^2+^ would affect growth or survival of *perM*::tn. Strains were cultured in nominally Mg^2+^-free Sauton’s minimal media, and supplemented with Mg^2+^ at a range of concentrations up to 2000 μM, the normal concentration in Sauton’s media (Fig. 3C). Wt Mtb died in nominally Mg^2+^-free media, but survived and replicated at Mg^2+^ concentrations of 25 μM and higher. In contrast, *perM*::tn exhibited death, observed by decreasing CFU counts, and lysis, observed by decreasing absorbance, at Mg^2+^ concentrations 100 μM and below. At 250 and 500 μM Mg^2+^, *perM*::tn replicated, but at a slower rate than wt and the complemented mutant.

The requirement for additional Mg^2+^ was specific, as other cations, including Mn^2+^, Ca^2+^, Zn^2+^, and Fe^3+^, could not restore growth of *perM*::tn in reduced (100 or 250 μM) Mg^2+^ media (S3 Fig.). For further experiments, Mtb was grown in modified Sauton’s media containing 250 or 500 μM Mg^2+^ (“reduced” Mg^2+^), concentrations at which *perM*::tn displayed a growth defect without apparent death or lysis, or 2000 μM (“high”) Mg^2+^.

Within the IFN-γ-activated macrophage, Mtb is subject to numerous stresses, including low pH, reactive nitrogen intermediates, reactive oxygen species, and nutrient limitation [29,30], and it has been postulated that Mg^2+^ restriction may be an additional stress encountered by intraphagosomal pathogens including Mtb [23,31,32]. The inability of *perM*::tn to replicate and survive in low Mg^2+^ raised the possibility that the persistence defect *in vivo* might follow depletion of intraphagosomal Mg^2+^ in activated macrophages. We infected resting and IFN-γ-activated macrophages with wt, *perM*::tn and the complemented mutant following growth in high (2 mM) and reduced (250 μM) Mg^2+^. The mutant displayed a growth defect in resting macrophages, which was larger when it was pre-cultured in reduced magnesium. Survival of *perM*::tn in IFN-γ-activated macrophages was impaired in comparison to wt and complemented mutant, but only following pre-culture in reduced magnesium (S4 Fig.). We examined whether *perM*::tn was more susceptible than wt Mtb to a range of stresses *in vitro*, including exposure to hydrogen peroxide, lysozyme, detergent, acidified sodium nitrite, free fatty acid, zinc, and copper, as well as carbon starvation, iron depletion, and a multi-stress assay combining a fatty acid carbon source, reduced pH, hypoxia, and sodium nitrite (S5 Fig.). The mutant survived at wt levels under all conditions, indicating that *perM*::tn does not have a general viability defect, but rather, appears to be specifically vulnerable to reduced Mg^2+^.

The increased Mg^2+^ requirement of *perM*::tn suggested a possible role for PerM in Mg^2+^ transport. However, analysis of total Mg^2+^ content by inductively coupled mass spectrometry (ICP-QQQ) showed no significant differences in strains grown in either high (2000 μM) or reduced (250 and 25 μM) Mg^2+^ (S6 Fig.), suggesting that PerM is not required for Mg^2+^ acquisition. Given our data, along with work in *Salmonella* suggesting a redundancy of Mg^2+^ transporters that ensures significant Mg^2+^ uptake [33], the persistent defect of *perM*::tn is unlikely the result of impaired Mg^2+^ transport.

In the context of the host response, infection of mouse macrophages with Mtb pre-grown in reduced (500 μM) Mg^2+^ media resulted in a 2-fold increase in TNF-α production by macrophages infected with *perM*::tn, but not wt or complemented strains (Fig. 3D). This suggests that either the immunostimulatory component(s) of *perM*::tn are more highly produced, secreted, or shed in reduced Mg^2+^; or that Mtb growth in reduced Mg^2+^ leads to increased exposure of these components to macrophage pattern recognition receptors and induction of a proinflammatory response.

### Increased expression of cell division and cell wall biosynthesis genes in *perM*::tn in reduced Mg^2+^

**Fig 4:**
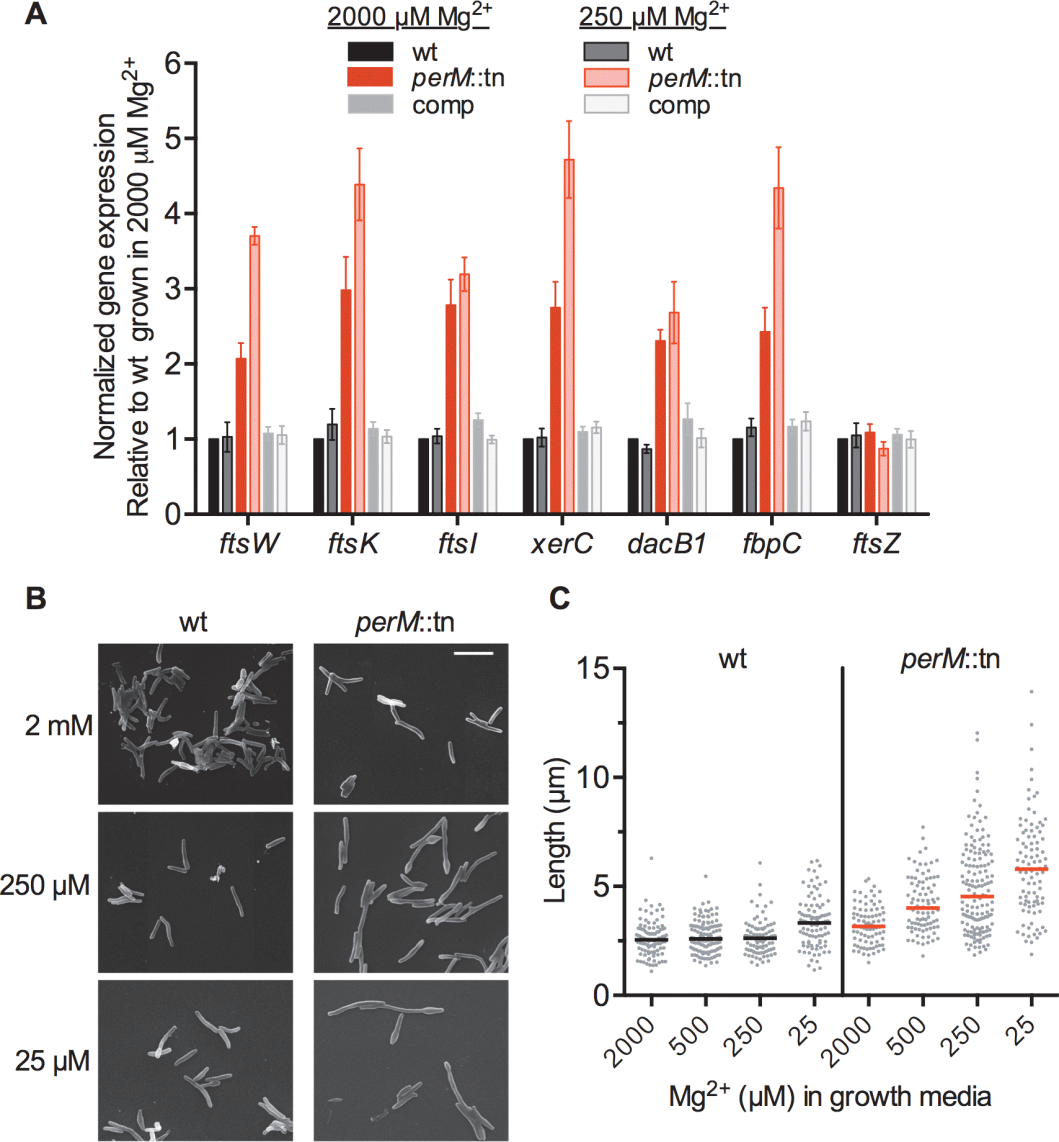
*PerM*::tn exhibits an Mg^2+^-dependent cell division defect. (**A**) mRNA levels for selected genes were compared by quantitative real time PCR. RNA was isolated from Mtb grown for 5 days in Sauton’s media containing 2000 or 250 μM Mg^2+^. mRNA levels were normalized to expression of the housekeeping gene *sigA*, then standardized to the wt sample grown in 2000 μM Mg^2+^ from the same experiment. Data are means ± SEM from five independent experiments. Data correlate with results obtained by microarray analysis. (**B**) Mtb was grown for 5 days at the indicated Mg^2+^ concentrations and imaged by scanning electron microscopy. Bar, 5 μm. (**C**) Lengths of bacteria from (B). Bar indicates median length. At least 80 bacteria were measured for each strain and concentration.

To gain insight into the function of PerM, we compared the transcriptomes of wt and perM::*tn* Mtb grown in high (2000 μM) and reduced (250 μM) Mg^2+^ in three independent experiments, using a p-value of 0.05 and 2-fold cutoff to identify differentially regulated genes. In reduced Mg^2+^, 41 genes were differentially expressed between strains, all of which except one were upregulated in the mutant (Table 1). Sixteen of these genes are annotated with predicted or possible roles in cell division and/or cell wall biosynthesis. Upregulation of a subset of these genes was confirmed by qRTPCR analysis (Fig. 4A).

**Table 1:** Transcriptome comparisons of wt and *perM*::tn in reduced Mg^2+^.

Rv #	Gene	FC	Description	Process	
Rv2894c*	xerC	3.8	Tyrosine integrase/recombinase	Cell division	
Rv2927c		3.4	Conserved hypothetical protein Possible cell division initiation protein	Cell division	
Rv2166c		3.3	Conserved hypothetical protein Ortholog of methyltransferase MraZ	Cell division	
Rv2165c	mraW	3.2	S-adenosyl-methyltransferase	Cell division	
Rv2164c*		3.2	Conserved proline rich membrane protein Possible cell division protein FtsL	Cell division	
Rv2748c*	ftsK	3.0	Cell division protein	Cell division	
Rv2154c	ftsW	2.7	Cell division protein	Cell division	
Rv2163c	pbpB/ftsI	2.6	Penicillin-binding membrane protein	Cell division	
Rv2147c		2.6	Conserved hypothetical protein Ortholog of cell division protein sepF	Cell division	
Rv2864c*		3.5	Penicillin-binding lipoprotein Possible transpeptidase PbpD	Cell wall biosynthesis	
Rv2525c		2.8	Conserved hypothetical protein	Cell wall biosynthesis	
Rv0129c	fbpC	2.7	Secreted fibronectin-binding protein C Possible trehalose mycolyltransferase	Cell wall biosynthesis	
Rv3330	dacB1	2.7	Penicillin-binding protein	Cell wall biosynthesis	
Rv1433		2.6	Hypothetical exported protein Possible L,D-transpeptidase LtdD	Cell wall biosynthesis	
Rv3717		2.5	Conserved hypothetical protein Ortholog of N-acetylmuramoyl-L-alanine amidase	Cell wall biosynthesis	
Rv0519c		2.3	Conserved membrane protein Possible mycolyltransferase	Cell wall biosynthesis	
Rv1251c		2.2	Conserved hypothetical protein Ortholog of RecB family nuclease	DNA replication/repair	
Rv1697		4.4	Conserved hypothetical protein Ortholog of thiamin pyrophosphokinase	Metabolism	
Rv1130		4.1	Ortholog of 2-methylcitrate dehydratase PrpD	Metabolism	
Rv3414c	sigD	2.6	Alternative RNA polymerase sigma factor	Regulatory	
Rv2720	lexA	2.8	Repressor protein	Regulatory	
Rv2034		2.2	ArsR-type repressor protein	Regulatory	
Rv3267		2.2	Conserved hypothetical protein Possible transcriptional regulator	Regulatory	
Rv1129c		2.2	Transcriptional regulator	Regulatory	
Rv0232		-2.1	Transcriptional regulator, tetR/acrR-family	Regulatory	
Rv1698*	mctB	3.6	Outer membrane channel	Transport	
Rv1672c		2.1	Conserved membrane transport protein	Transport	
Rv1566c		2.9	Invasion-associated protein	Virulence	
Rv3810	pirG/ erp	2.6	Exported repetitive protein	Virulence	
Rv0996*		3.7	Conserved membrane protein		
Rv2255c		3.7	Hypothetical protein		
Rv2254c		3.4	Membrane protein		
Rv1435c		3.2	Conserved glycine, proline and valine-rich secreted protein		
Rv3209*		2.8	Conserved proline and threonine rich protein Ortholog of MmpS3 membrane protein		
Rv2253		2.6	Secreted protein		
Rv2256c		2.5	Conserved hypothetical protein		
Rv1157c		2.4	Conserved alanine and proline rich protein		
Rv1158c		2.4	Conserved alanine and proline rich protein		
Rv3413c		2.2	Hypothetical alanine and proline rich protein		
Rv3054c		2.1	Conserved hypothetical protein		
Rv3096		2.1	Conserved hypothetical protein		
Rv3258c		2.0	Conserved hypothetical protein	

Genes listed were differentially expressed at least 2-fold in *perM*::tn compared to wt grown for 5 days in media supplemented with 250 μM Mg^2+^. Fold change values are averages of three independent experiments, *P*<0.05. Annotations adapted from TB Database (tbdb.org), TubercuList (tuberculist.epfl.ch) and PATRIC (patricbrc.org). FC, fold change in *perM*::tn compared to wt. Genes also regulated greater than 2-fold between strains in 2000 μM Mg^2+^ are marked with *.

Cell division genes more highly expressed in the mutant compared to wt under reduced Mg^2+^ included *ftsK* and *xerC*, involved in chromosome segregation; *ftsI*, necessary for peptidoglycan crosslinking during division; and *ftsW*, whose product likely translocates peptidoglycan precursors across the cell membrane [34] and interacts with both FtsI as well as cell division initiator FtsZ in mycobacteria [35]. Also upregulated in the mutant were genes encoding four putative penicillin binding proteins (FtsI, DacB1, Rv2864c, and Rv1433), enzymes which carry out the transpeptidation necessary for crosslinking of cell wall peptidoglycan strands; Rv3717, a possible peptidoglycan amidase with a role in cell wall remodeling; and Rv0519c, a possible mycolyltransferase involved in mycolic acid processing [36]. Secreted fibronectin-binding protein C (FbpC), a possible trehalose mycolyltransferase thought to have both antigenic and cell wall biosynthesis roles, also showed increased expression in the mutant. Notably, expression of *ftsZ*, encoding the cytosolic, tubulin-like initiator of cell division, was not increased in the mutant at either Mg^2+^ concentration, nor was expression of genes in the cell wall biosynthetic gene cluster (*rv3779-rv3809c*) contributing to mycolic acid, arabinogalactan, and LAM synthesis [37], pointing towards a specific response rather than a global induction of all cell division and cell wall biosynthesis genes in the mutant.

Seven genes were upregulated in the mutant compared to wt in both high and reduced Mg^2+^, with more pronounced differences in expression between strains in reduced Mg^2+^ (Tables 1, S1), suggesting that Mg^2+^ reduction exacerbates differential transcriptional responses that are also present in high Mg^2+^.

Comparison of gene expression in wt Mtb in reduced versus high Mg^2+^ revealed only two genes meeting the 2-fold cutoff: *pe20* was upregulated in reduced Mg^2+^ and *fadD5* was downregulated (S2 Table). This transcriptional response was far less pronounced than that previously identified consisting of 24 genes differentially regulated in wt Mtb grown in media with or without Mg^2+^ [22], suggesting that the transcriptional response to Mg^2+^ starvation in wt Mtb was not triggered at 250 μM Mg^2+^, used in our experiment. The gene expression pattern of *perM*::tn in 250 μM Mg^2+^ (S2 Table) did not resemble Mg^2+^-starved wt Mtb [22], contrary to what might be expected if Mg^2+^ uptake were impaired in the mutant.

### PerM accumulates at the septum and is necessary for division in reduced Mg^2+^

The increased expression of cell division and cell wall biosynthesis genes in the mutant suggested a possible defect in these processes. To examine the impact of *perM* disruption on cell morphology, Mtb was grown in a range of Mg^2+^ concentrations, fixed, and imaged by scanning electron microscopy (SEM). *PerM*::tn exhibited Mg^2+^-dependent defects in morphology and division. Median cell length increased as the concentration of Mg^2+^ decreased, and some mutant bacilli exhibited bulging at the poles in reduced Mg^2+^ (Fig. 4B,C).

**Fig 5:**
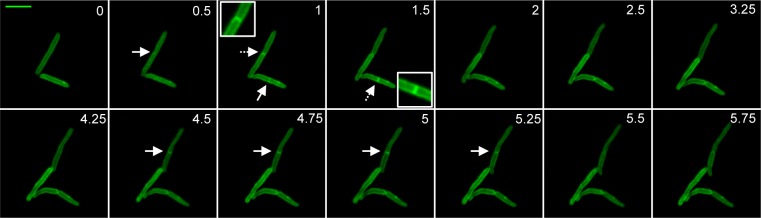
PerM accumulates at the septum during cell division. Representative image series of replicating *M*. *smegmatis* expressing PerM-GFP. Arrows point to septa. Inset corresponds to septum indicated by a dashed arrow. Numbers indicate time in hours. Scale bar, 4 μm.

To examine localization of PerM, GFP-tagged Mtb PerM protein was expressed in wt *Mycobacterium smegmatis*, a non-pathogenic species closely related to Mtb and itself containing an PerM homolog with 73% identity. Mtb requires containment within a biosafety level 3 facility, which prevented us from performing live cell imaging experiments in Mtb. Live cell imaging of recombinant *M*. *smegmatis* revealed that PerM_Mtb_ localized to the membrane and it accumulated at the mid-cell division site (Fig. 5), similar to mycobacterial cell division proteins, such as FtsI and FtsZ, as well as peptidoglycan synthesis enzymes, such as penicillin binding protein 1 [38–40].

### *PerM*::*tn* is hypersensitive to beta lactam antibiotics

We next compared sensitivity of *perM*::tn and wt Mtb to a range of compounds targeting cell wall biosynthesis, as well as drugs with other established targets. The majority of compounds assayed exhibited a similar minimum inhibitory concentration (MIC) in wt and *perM*::tn (Table 2 and S7 Fig.), with a shift of 2-fold or less considered insignificant. However, *perM*::tn was acutely sensitive to growth inhibition by β-lactam antibiotics, which target penicillin binding proteins that carry out the transpeptidation reaction resulting in crosslinking of cell wall peptidoglycan, a final step in peptidoglycan synthesis. The shift in MIC was most pronounced for cephalexin and piperacillin, β-lactams that specifically inhibit FtsI, the transpeptidase required for peptidoglycan crosslinking during bacterial cell division [41–43]. β-lactamase activities in wt and *perM*::tn were not significantly different (S3 Table) excluding the possibility that impaired β-lactamase activity caused the mutant’s increased susceptibility to β-lactams. Notably, the MICs of vancomycin and D-cycloserine, which inhibit earlier steps in peptidoglycan synthesis than do β-lactams, were similar for wt and *perM*::tn. Furthermore, there was little to no shift in MIC of isoniazid and ethambutol, which inhibit production of other cell wall components (mycolic acids and arabinogalactan, respectively), indicating that the *perM*::tn is not broadly hypersusceptible to interference with cell wall biosynthesis.

**Table 2:** *PerM*::tn is highly sensitive to β-lactam antibiotics.

Compound	Target	wt	*perM*::tn	FC
Piperacillin	Peptidoglycan	156	4.88	32
Cephalexin	Peptidoglycan	19.5	0.61	32
Ampicillin	Peptidoglycan	12.5	0.78	16
Meropenem	Peptidoglycan	1	0.12	8
Rifampicin	RNA polymerase	3.12	0.78	4
Polymyxin B	Membrane	12.5	3.12	4
DCCD	ATP synthase	500	250	2
Vancomycin	Peptidoglycan	0.62	0.31	2
D-Cycloserine	Peptidoglycan	6.25	3.12	2
Streptomycin	Protein synthesis	0.62	0.31	2
Chloramphenicol	Protein synthesis	50	25	2
Isoniazid	Mycolic acid	15.6	7.81	2
Ethambutol	Arabinogalactan	3.12	3.12	1

Minimum inhibitory concentration (MIC) of various drugs against wt and *perM*::tn Mtb. MIC_90_ values in μg/mL, determined by minimum concentration at which OD_580_ was less than 10% that of untreated control. FC, fold change reduction of *perM*::tn MIC compared to wt MIC.

## Discussion

This work implicates a novel mycobacterial membrane protein in cell division and demonstrates its requirement for Mtb persistence *in vivo*. The persistence defect of the PerM mutant is one of the most dramatic *per* phenotypes observed to date, and to our knowledge the first noted in a mutant of an Mtb membrane protein. Global gene expression profiling revealed increased expression of cell division and cell wall biosynthesis genes in the mutant, and these increases exacerbated during growth in reduced Mg^2+^. Several additional observations support the hypothesis that PerM plays a role in cell division. First, the mutant elongated in reduced Mg^2+^, with additional morphological changes at very low Mg^2+^. Second, the mutant exhibited hypersusceptibility to β-lactam antibiotics, which inhibit the enzymes necessary for crosslinking of cell wall peptidoglycan. In particular, the mutant was hypersusceptible to piperacillin and cephalexin, β-lactams that specifically target the cell division-associated peptidoglycan transpeptidase, FtsI [42–44]. Third, PerM localized to the mid-cell region in *M*. *smegmatis*, similarly to previously studied mycobacterial proteins involved in cell division and peptidoglycan biosynthesis [38–40]. The mutant hyperstimulated mouse macrophages *ex vivo*, a phenotype exacerbated after culture in reduced Mg^2+^, which may be related to shedding of cell wall components during a compromised cell division process.

The inability of *perM*::tn to replicate and survive at low Mg^2+^ suggested that PerM may play a role in Mg^2+^ acquisition, could be necessary for the adaptive response of Mtb to low Mg^2+^, or that Mg^2+^ might serve a compensatory function to mask physiological defects caused by the absence of PerM. We examined the first possibility by ICP-QQQ analysis, which revealed *perM*::tn and wt Mtb to contain the same total Mg^2+^, even when grown in reduced Mg^2+^ media. Furthermore, gene expression data from the mutant showed a regulation pattern distinct from that of Mg^2+^-starved wt Mtb [22]. The second possibility, that PerM is a component of the bacterial response to low Mg^2+^, is similarly not supported by the gene expression profile of wt Mtb grown in low Mg^2+^ [22]. However, it is possible that PerM, constitutively expressed, is required for a successful adaptive response to Mg^2+^ starvation, perhaps through interaction with Mg^2+^ response proteins. Future protein interaction studies may shed light on this question.

Our work supports the third possibility, that Mg^2+^ serves a compensatory function in the mutant through stabilization of a weakened cell envelope; in particular, our data suggest that the mutant cell envelope may be especially vulnerable during cell division. While the role of Mg^2+^ in cell wall stability is widely acknowledged, the mechanism by which this occurs is not entirely clear. In *Salmonella*, outer membrane permeability decreased in high Mg^2+^, and a *phoP Salmonella* mutant with lipopolysaccharide alterations displayed increased permeability and susceptibility to numerous antibiotics in low Mg^2+^, but behaved like wt *Salmonella* when Mg^2+^ was high [45]. This suggests a role for Mg^2+^ in stabilizing the outer membrane, perhaps through interaction with negatively-charged lipopolysaccharide. In the Gram-positive *B*. *subtilis*, which lacks an outer membrane, high Mg^2+^ partially suppressed the growth defect of a mutant lacking teichoic acid suggesting that Mg^2+^ might be able to compensate for loss of teichoic acid in the cell wall [46]. On the other hand, high concentrations of Mg^2+^ may serve to stabilize an otherwise vulnerable peptidoglycan sacculus. *B*. *subtilis* mutants lacking MreB, RodB and PonA—proteins thought to be involved in peptidoglycan synthesis—display morphological and growth defects that were rescued by high Mg^2+^ [47–49]. Of note, peptidoglycan synthesis decreased and peptidoglycan precursors accumulated in Mg^2+^-deprived *B*. *subtilis* [50], and in *Salmonella*, lipid A acylation increased in response to Mg^2+^ deprivation [20], suggesting that the influence of Mg^2+^ on peptidoglycan integrity may occur by several mechanisms, both structural and regulatory. It has also been proposed that Mg^2+^ might affect the degree of peptidoglycan crosslinking that occurs; stabilize or regulate important cell-wall synthases or hydrolases; or serve to stiffen the cell envelope [45,51].

The upregulation of cell division genes in *perM*::tn, combined with the hypersensitivity of the mutant to specific inhibitors of FtsI, suggests a role for PerM in peptidoglycan synthesis or remodeling during cell division. The *perM* mutant was not hypersusceptible to all peptidoglycan synthesis inhibitors: the MICs of vancomycin and cycloserine were similar for mutant and wt Mtb. Cycloserine, an analog of D-alanine, blocks synthesis of cytoplasmic peptidoglycan precursors [52], while vancomycin prevents both the early transglycosylation step necessary for incorporation of peptidoglycan monomer into the sacculus, as well as the final crosslinking of peptidoglycan by transpeptidases [53]. The specific vulnerability of *perM*::tn to β-lactams, which target the transpeptidation step, suggests that PerM may play a role in late peptidoglycan biosynthesis during cell division. Interestingly, a conditional mutant of *ripA*, which encodes an essential mycobacterial peptidoglycan hydrolase, was similarly hypersusceptible to a β-lactam, carbenicillin, but not to cycloserine following *ripA* depletion [54].

While its specific mechanism of action remains to be determined, it is plausible that PerM, as an integral membrane protein with 10 transmembrane helices, could serve a structural role, recruiting or anchoring key cell division proteins, such as peptidoglycan transpeptidases or hydrolases, to the division site. It may serve to bridge cytoplasmic proteins, such as FtsZ or early peptidoglycan synthesis machinery, with cell division proteins in the periplasm, or it could be involved in transport of cell envelope components.

The *perM* mutant withstood numerous stresses *in vitro*, including reactive oxygen and nitrogen species, cell wall-perturbing detergent, and carbon starvation, showing a specific vulnerability to a low-Mg^2+^ environment. Surprisingly, the *perM* mutant was not more susceptible than wt to exposure to arachidonic acid at pH 5.5, despite its sensitivity to Tween-80 at pH 4.5, which suggested that free oleic acid might be toxic to the mutant at low pH. It is plausible that oleic acid released from Tween-80 and arachidonic acid cause toxicity by different mechanisms. In addition, the lower pH of the Tween-80 containing medium may have contributed to the enhanced killing of the mutant.

Survival of *perM*:tn in IFN-γ activated macrophages was impaired, when the bacteria were pre-grown in reduced Mg^2+^. IFN-γ activated, Mtb infected macrophages have a limited lifespan *ex vivo*, which prevented extending the time course of the *ex vivo* infection to better mimic the mouse infection. It is possible that pre-growth in reduced Mg^2+^ has the same impact as replication in the acute phase of mouse infection, but interpretation of the *ex vivo* macrophage infection data is difficult and does not allow direct conclusions about the intraphagosomal availability of Mg^2+^.

Previous work revealed that macrophage activation by IFN-γ results in changes in the intraphagosomal concentrations of several metals, but Mg^2+^ was not measured [55]. The *Salmonella*-containing phagosome was estimated to contain 10 to 50 μM Mg^2+^, based on strong induction of the Mg^2+^-regulated *mgtB* gene in *Salmonella* in both low Mg^2+^ media and upon uptake by mammalian cells [56,57]; however, measurement of intraphagosomal Mg^2+^ using nanosensor particles showed the concentration to be approximately 1 mM in the first two hours of infection [58]. The intraphagosomal concentration of Mg^2+^ *in vivo*, after days or weeks of Mtb infection, remains a topic of speculation. Our work lends support to the hypothesis of an Mg^2+^-depleted environment in the Mtb-containing activated macrophage *in vivo*. Unfortunately, measuring intraphagosomal Mg^2+^ concentrations is extremely challenging. Purification of Mtb infected phagosomes is difficult and it is unknown if the purification process alters phagosomal ion concentrations. Fluorescent Mg^2+^ reporters exhibit much higher affinity for Ca^2+^ and also bind Zn^2+^, while PEPPLE (probe encapsulated by biologically localized embedding) technology suffers from low magnesium affinity [59]. Future development of novel and better sensors for magnesium is required to overcome these obstacles.

The PerM mutant stimulated a hyperinflammatory cytokine response in infected macrophages *ex vivo*. While we did not detect elevated cytokine levels in lungs of mice infected with the *perM* mutant compared to wt-infected mice, we cannot rule out the possible contribution of a hyperinflammatory response to the persistence defect. The cytokine measurements might have been confounded by differences in bacterial loads, and even a small, perhaps difficult to quantify, difference in the host immune response could synergize with Mg^2+^ restriction to result in killing of *perM*::tn; or the response may be localized, with lesion-centric inflammation contributing to killing *perM*::*tn*, but little impact on total cytokine levels in the lungs.

Growth of Mtb in reduced Mg^2+^ prior to macrophage infection resulted in an augmented response to the mutant, but no difference in response to wt Mtb, suggesting that cell wall instability of the mutant may contribute to the hyperinflammatory phenotype. In light of other evidence linking PerM to cell division, it is plausible that the mutant sheds multiple components of the cell wall during a stalled or otherwise compromised division process, resulting in increased stimulation of macrophage response pathways.

The bacterial cell wall is the target of many drugs in current use. The remarkable sensitivity of *perM*::tn to cephalexin and piperacillin, antibiotics routinely and safely used in clinical practice, suggests an exciting possibility of PerM as a co-target. An inhibitor of PerM could potentially be used to sensitize Mtb to β-lactam antibiotics, extending their use to mycobacterial infections.

## Materials and Methods

### Ethics statement

Mouse studies were performed following National Institutes of Health guidelines for housing and care of laboratory animals and performed in accordance with institutional regulations after protocol review and approval by the Institutional Animal Care and Use Committee of Weill Cornell Medical College (protocol # 2008–0006, pH homeostasis in *Mycobacterium tuberculosis*).

### Bacterial strains and media

*PerM*::tn, the Mtb H37Rv transposon mutant of gene *perM* (*rv0955*) containing a ΦMycoMarT7 transposon insertion at nucleotide 701, was isolated in a screen for acid-sensitive mutants described previously [13]. Mtb strains were grown in a humidified incubator at 37°C with 5% CO_2_ in Sauton’s media with 0.05% Tween 80 or 0.05% tyloxapol; Middlebrook 7H9 medium (Difco) containing 0.2% glycerol, 0.5% bovine serum albumin, 0.2% dextrose, 0.085% NaCl, and 0.05% Tween 80; or Middlebrook 7H11 agar (Difco) containing 10% OADC supplement (Becton Dickinson) and 0.5% glycerol. Nominally magnesium-free Sauton’s media was prepared with 0.8 mM citric acid, 9 mM sodium citrate, 3 mM potassium phosphate, 30 mM L-asparagine, and 6% glycerol, chelated overnight with 20 g/L Chelex 100 resin (Bio-Rad), filtered to remove Chelex, supplemented with 0.2 mM ferric ammonium citrate and 5 μM zinc sulfate, and adjusted to pH 7.4. Before use, 0.05% Tween 80 and 2 mM MgCl_2_ were added unless otherwise indicated. We call this medium “nominally magnesium-free” as trace residual magnesium is likely present. Hygromycin B (50 μg/ml), kanamycin (15 μg/ml) and streptomycin (20 μg/ml) were included when required for selection.

### Complementation of *perM*::tn

*Rv0955* was PCR amplified from H37Rv genomic DNA and cloned behind the hsp60 promoter into a plasmid that integrates into the chromosomal phage integration attL5 site. For localization studies, GFP was fused to the C-terminus of Rv0955 and expressed from the hsp60 promoter on an integrative plasmid.

### Mouse infections

Female C57BL/6, or IFN-γ^-/-^ mice (Jackson Laboratory) were infected using an inhalation exposure system (Glas-Col) with early-log-phase Mtb to deliver approximately 100 bacilli per mouse. Bacterial numbers were enumerated by plating serial dilutions of lung or spleen homogenates on 7H11 agar plates for CFU. Upper left lung lobes were fixed in 10% buffered formalin, embedded in paraffin and stained with hematoxylin and eosin.

### Macrophage infection experiments

Bone marrow derived macrophages were harvested and differentiated as previously described [13] and seeded at 4x10^6^ cells/mL, with or without 50 ng/mL murine IFN-γ (R&D Systems). Approximately sixteen hours later, macrophages were infected at a multiplicity of infection (MOI) of 0.1 with a single cell suspensions of log-phase Mtb grown for 6 days in 250 or 2000 μM MgCl_2_. Monolayers were washed with PBS 4 hours post-infection to remove extracellular bacteria. After 4 hours, 3 days, or 6 days, macrophages were lysed with 0.5% Triton X-100 and bacteria were enumerated by plating serial dilutions on 7H11 agar plates. Half of the media in each well was replaced with fresh media after 3 days.

### Measurement of cytokine production by infected macrophages *ex vivo* and *in vivo*

Bone marrow derived macrophages from C56BL/6 or TLR2^-/-^ mice (Jackson Laboratories) were harvested and differentiated as previously described [13]. Immortalized macrophage cell lines from wild type, and Nod1/2^-/-^ mice [60] were a gift from M. A. Kelliher at the University of Massachusetts. Macrophages were seeded at 4x10^5^ cells/ml (wt macrophages) or 6x10^5^ cells/ml (knockout macrophages). After 16 hours, they were infected at the indicated MOI with a single cell suspension of log-phase Mtb. For experiments using dead bacteria, Mtb was fixed in 10% formalin for 16 hours, washed twice in PBS, and added to Mtb at an MOI of 20. For exposure of macrophages to Mtb-conditioned culture media, Mtb was grown for 8 days in detergent-free Sauton’s media containing 2 mM MgCl_2_, then culture supernatant was passed through a 0.2 μm filter, concentrated approximately 10-fold in Amicon Ultra-15 Centrifugal Filter Units (Millipore), and added to macrophages at a volume equivalent to 10 μg protein. Supernatants were collected after 24 hours, passed through a 0.2 μm filter, and stored at -80°C. Cytokine levels were quantified using BD OptEIA ELISA kits for mouse TNF or IL-12p40 (BD Biosciences), or a multiplex ELISA Mouse ProInflammatory 7-Plex Tissue Culture Kit (Meso Scale Discovery). Tissue processing, RNA isolation, and real-time PCR were performed as previously described [61].

### *In vitro* stress susceptibility assays

Mtb was grown to log phase in Sauton’s media containing 2000 μM MgCl_2_ prior to each experiment. Single cell suspensions were prepared in assay medium by centrifugation at 800 rpm for 12 minutes, then diluted to OD 0.02–0.05 and incubated in the following conditions: 3 days in pH 4.5 in media containing 0.05% Tween 80 or tyloxapol; 5 weeks in PBS with 0.05% tyloxapol; 24 hours in 7H9 media with 0.05% Tween 80 and 2.5 mg/ml lysozyme; 5 hours in 7H9 media with 0.05% Tween 80 and 0.1% SDS; 3 days in 7H9 media at pH 5.5 with 0.05% tyloxapol and 5 mM NaNO_2_; 3 days in 7H9 media at pH 5.5 with 0.05% tyloxapol and 50 μM arachidonic acid; 3 hours in 7H9 media containing 10 mM H_2_O_2_. For the multi-stress survival assay, Mtb was incubated in 1% oxygen for 14 days in modified Sauton’s media at pH 5.5 containing 0.05% tyloxapol, 0.05% butyrate, 0.5 mM sodium nitrite, 2000 μM MgCl_2_, and without glycerol. The exposure times to different stress conditions were selected so that viability of wt Mtb was reduced by approximately 5- to 10-fold. For conditions in which wt Mtb survived without significant or very slow death (carbon starvation, multi-stress model) extended incubation times were chosen. To determine viability, serial dilutions of cultures were plated on 7H11 plates.

### Cation dose response experiments

Mtb was grown to log phase, washed twice in assay medium, and diluted to OD_580_ 0.02 in plates containing two-fold serial dilutions of MgCl_2_, MgSO_4_, ZnCl_2_, MnCl_2_, CaCl_2_, CuCl_2_, or ferric ammonium citrate. For experiments testing various cations as substitutes for Mg^2+^, a basal level of either 100 or 250 μM MgCl_2_ was added as indicated. For experiments involving ZnCl_2_ or ferric ammonium citrate, modified Sauton’s medium was prepared without the respective cation.

### ICP-QQQ analysis

Mtb was washed twice in nominally Mg^2+^-free Sauton’s media, diluted to OD 0.1 in Sauton’s media containing 250 or 2000 μM added MgCl_2_. After 5 days, cultures were washed twice in PBS with 0.05% Tween 80. To determine the impact of very low Mg^2+^, cultures were grown in Sauton’s media containing 2000 μM added MgCl_2_ until mid-log phase, then washed twice in nominally Mg^2+^-free Sauton’s media and incubated in Sauton’s media containing 25 μM added MgCl_2_. Pellets were collected at 0 hour, 3 hours and 10 hours post inoculation. After normalizing for biomass, pellets were heated at 80°C for 1 hour to kill Mtb, then resuspended in 200 μL 70% nitric acid, trace element grade (Fisher) and heated at 80°C for 2h before ICP-QQQ processing and analysis. Samples were analyzed on an Agilent 8800 ICP-QQQ running in MS/MS mode. Instrument daily performance qualification and method specific tuning was achieved by the expert AutoTune function of the MassHunter software (B.01.02). Typical sample introduction parameters for direct injection were used; RF Power 1550W, sample depth 8 mm, carrier gas 0.95 L/min, and dilution gas was set at 0.15 L/min. These parameters resulted in an oxide ratio of 0.8% (CeO/Ce). Prior to analysis, samples were diluted to a final volume of 2 ml and analyzed against multi-element external calibration standards (Agilent, Wilmington, DE). NIST 1643e was used as a standard reference material for calibration verification and monitor any possible drift during the analytical run.

### RNA isolation and gene expression analysis

Mtb was grown standing for 5 days in Sauton’s media containing 250 or 2000 μM MgCl_2_ and 0.05% Tween 80. Flasks were shaken for 5 hours prior to harvest. Cultures were mixed with an equal volume of GTC buffer containing guanidinium thiocyanate (4 M), sodium lauryl sulfate (0.5%), trisodium citrate (25 mM), and 2-mercaptoethanol (0.1 M) and pelleted by centrifugation. Bacterial RNA was isolated as previously described [62]. For microarray experiments, RNA was labeled using a Low Input Quick Amp Labeling Kit (Agilent). Microarrays were custom-designed (Genotypic Technology, Bangalore, India). Analysis was performed using Agilent GeneSpring software. The complete Microarray data sets have been submitted to the Gene Expression Omnibus (GEO) database.

For gene expression analysis by quantitative real-time PCR, cDNA was generated using MuLV Reverse Transcriptase (Invitrogen) and quantified using Roche Light Cycler 480 Real-Time PCR System with primers and TaqMan probes designed using Primer3 (http://bioinfo.ut.ee/primer3-0.4.0). Primer and probe sequences are available upon request.

### Electron microscopy and length measurement

Mtb was grown for 5 days in Sauton’s media containing 25, 250, 500, or 2000 μM MgCl_2_ and 0.05% Tween 80 before fixation, processing, and imagining by scanning electron microscopy as previously described [13]. Cell lengths were measured using Adobe Photoshop software.

### Antibiotic sensitivity assays

Mtb was grown to early log phase and diluted to an optical density of 0.02 in Sauton’s medium containing 2 mM MgCl_2_ and 0.05% Tween 80. Bacteria were then exposed to twofold dilutions of piperacillin, cephalexin, ampicillin, meropenem, rifampicin, polymyxin B, DCCD, vancomycin, D-cycloserine, streptomycin, chloramphenicol, isoniazid, and ethambutol (Sigma-Aldrich). For assays of meropenem, DCCD, isoniazid, chloramphenicol, and rifampicin, all wells contained 0.5% DMSO. For the assay of cephalexin, all wells contained 4 mM NH_4_OH. The MIC was recorded as the minimum concentration at which growth, measured by optical density (OD_580_), was inhibited by at least 90%, as compared to a control containing no antibiotic, after approximately 2 weeks.

### Fluorescence microscopy

*M*. *smegmatis* expressing PerM-GFP was sealed in B04A microfluidic flow chamber plates (Cell Asic, part of EMD Millipore) and perfused with Middlebrook 7H9 broth at 37°C. Cells were visualized by fluorescence microscopy using an inverted Olympus IX-70 microscope equipped with a GFP filter set, a Photometrics CoolSnap QE cooled CCD camera, and an Insight SSI 7 color solid state illumination system. Snapshots were captured every 15 minutes.

### ß-lactamase activity assay

The chromogenic cephalosporin nitrocefin (Fisher) was used to assay ß-lactamase activity as previously described [63] in whole-cell lysates of Mtb saturated cultures grown in Sauton’s media with 2 mM MgCl_2_.

## Supporting Information

S1 Fig Genomic organization and predicted topology of Rv0955 (PerM). **(A)** Genomic organization of *perM* and neighboring genes. Genes depicted as arrows, with transposon insertion sites indicated by arrowheads. Two transposon mutants were identified with insertions at nucleotides 701 and 1106 of *perM*. In the current work the mutant with insertion at nucleotide 701 was used. **(B)** PerM topology predicted by the TMHMM server using a hidden Markov model [15]. Illustrated with TMRPres2D [16].
(TIFF)

S2 Fig Cytokine response *in vivo*. (**A**) qRTPCR measurement of total mRNA levels in lungs of infected mice, relative to ß-actin expression. (**B**) CFU from lungs analyzed in A. (**C**) IL-12 p40 content of lung homogenates of mice infected for 7, 14, or 21 with wt or *perM*::tn Mtb. IL-12 p40 was measured by ELISA. (**D**) CFU from lungs analyzed in C. The differences in CFU at week 1 and 2 post infection were not statistically significant. Data are means ± SD from 4 or 5 mice. * P = 0.026.
(TIFF)

S3 Fig Magnesium requirement of perM::tn cannot be met by substitution with other cations. (**A**) Growth of wt and *perM*::tn after 7 days in media with increasing Mg^2+^ concentrations. (**B**) Growth after 7 days in media containing 100 μM (open symbols) or 250 μM (closed symbols) Mg^2+^ and supplemented with increasing concentrations of Mn^2+^, Ca^2+^, Zn^2+^, or Fe^3+^. Growth expressed as percent OD_580_ of control culture, grown in 2 mM Mg^2+^.
(TIFF)

S4 Fig Growth and survival of *perM*::tn in macrophages. Mtb strains were grown for 6 days in 250 or 2000 μM Mg^2+^ prior to infection. CFU recovered from resting (**A**) and IFN-γ-activated (**B**) macrophages at the indicated time points. Data are means ± SD from triplicates cultures. *** P < 0.005
(TIFF)

S5 Fig PerM::tn exhibits wt-level resistance to numerous stresses. (**A**-**H**) Survival determined by CFU. Data are means ± SD of triplicate cultures. (**A**) Susceptibility to low pH. Mtb was incubated for 3 days at pH 4.5 in media containing 0.05% Tween-80 (left) or tyloxapol (right) as detergent. (**B**) Carbon starvation. Survival in phosphate buffered saline with 0.05% tyloxapol. (**C**) Susceptibility to lysozyme. Survival following exposure to 2.5 mg/ml lysozyme for 24 hours. (**D**) Susceptibility to detergent perturbation. Survival following exposure to 0.1% SDS for 5 hours. (**E**) Susceptibility to reactive nitrogen intermediates (RNI). Survival following exposure to 5 mM NaNO_2_ in pH 5.5 media for 3 days. (**F**) Susceptibility to free fatty acids. Survival following exposure to 50 μM arachidonic acid in pH 5.5 media for 4 hours. (**G**) Susceptibility to reactive oxygen species (ROS). Survival following exposure to 10 mM H_2_O_2_ for 3 hours. Dotted line indicates input CFU. (**H**) Survival in a multi-stress assay consisting of a fatty acid carbon source (0.05% butyrate), 1% oxygen, reduced pH (5.5), and 0.5 mM sodium nitrite. (**I**-**K**) Growth inhibition by treatment with (**I**) ZnCl_2_, (**J**) CuCl_2_, or (**K**) iron chelator 2,2-dipyridyl. OD_580_ expressed as a percent of untreated control.
(TIFF)

S6 Fig *PerM*::tn is not required for Mg^2+^ acquisition. (**A**) Inductively coupled plasma mass spectrometry (ICP-QQQ) measurement of total Mg^2+^ content of Mtb pellets after 6 days of growth in either reduced (250 μM) or high (2000 μM) Mg^2+^. (**B**) ICP-QQQ of total Mg^2+^ content of Mtb pellets before (t0) and after 3 and 10 hour incubation in 25 μM Mg^2+^. Samples were normalized for biomass. Data are means ± SD of three replicates and data in A are representative of two independent experiments.
(TIFF)

S7 Fig *PerM*::tn is hypersusceptible to β-lactam antibiotics. Dose response curves showing growth of Mtb cultures after exposure to the indicated drug for 7 days. Data are averages of two replicates and representative of at least two independent experiments.
(TIFF)

S1 Table Transcriptome comparison of wt and *perM*::tn in high Mg^2+^. Genes listed were differentially expressed with a fold change ≥ 2.0 and *P*<0.05 in *perM*::tn compared to wt grown for 5 days in media supplemented with 2000 μM Mg^2+^. Fold change values are averages of three independent experiments, *P*<0.05. Annotations adapted from TB Database (tbdb.org), TubercuList (tuberculist.epfl.ch), and PATRIC (patricbrc.org). FC, fold change in *perM*::tn compared to wt. Genes also regulated greater than 2-fold between strains in 250 μM Mg^2+^ are marked with *.
(PDF)

S2 Table Mg^2+^-regulated genes. Global transcriptome profiling of log-phase cultures by microarray analysis. Genes listed were differentially expressed with a fold change ≥ 2.0 and *P*<0.05 in in reduced (250 μM) Mg^2+^ media, compared to high (2000 μM) Mg^2+^ media, in the indicated strain. Fold change values are averages of three independent experiments, *P*<0.05.
(PDF)

S3 Table ß-lactamase activity assays on Mtb whole cell lysates. Nitrocefin activity is expressed as μg nitrocefin hydrolyzed min^-1^ (mg total protein)^-1^ ± SD. The differences between the strains were not statistically significant. Each assay was performed with triplicate cultures.
(PDF)
