# Supplementary material for: Disruption of an M. tuberculosis Membrane Protein Causes a Magnesium-dependent Cell Division Defect and Failure to Persist in Mice
Source: PLoS Pathog. 2015 Feb 6;11(2):e1004645. doi: 10.1371/journal.ppat.1004645 (PMC4450064; doi:10.1371/journal.ppat.1004645)
Supplement: S1 Table — Genes listed were differentially expressed with a fold change ≥ 2.0 and P<0.05 in perM::tn compared to wt grown for 5 days in media supplemented with 2000 μM Mg2+. Fold change values are averages of three independent experiments, P<0.05. Annotations adapted from TB Database (tbdb.org), TubercuList (tuberculist.epfl.ch), and PATRIC (patricbrc.org). FC, fold change in perM::tn compared to wt. Genes also regulated greater than 2-fold between strains in 250 μM Mg2+ are marked with *. (PDF) [file ppat.1004645.s008.pdf]

Rv #	Gene	FC	Description	Process
Rv2864c*		2.6	Penicillin-binding lipoprotein<br>Possible transpeptidase PbpD	Cell wall biosynthesis
Rv0996*		2.6	Conserved membrane protein	
Rv3074		2.5	Conserved hypothetical protein<br>Ortholog of HNH nuclease	DNA replication/repair
Rv1698*	*mctB*	2.4	Outer membrane channel	Transport
Rv3209*		2.2	Conserved proline & threonine rich protein<br>Ortholog of MmpS3 membrane protein	
Rv1378c		2.2	Conserved hypothetical protein<br>Ortholog of HNH nuclease	DNA replication/repair
Rv2164c*		2.2	Conserved proline rich membrane protein<br>Possible cell division protein FtsL	Cell division
Rv2748c*	*ftsK*	2.1	Cell division protein	Cell division
Rv2894c*	*xerC*	2.1	Tyrosine integrase/recombinase	Cell division
Rv3370	*dnaE2*	2.0	DNA polymerase III alpha chain dnaE2	DNA replication/repair
