# Supplementary material for: Disruption of an M. tuberculosis Membrane Protein Causes a Magnesium-dependent Cell Division Defect and Failure to Persist in Mice
Source: PLoS Pathog. 2015 Feb 6;11(2):e1004645. doi: 10.1371/journal.ppat.1004645 (PMC4450064; doi:10.1371/journal.ppat.1004645)
Supplement: S2 Table — Global transcriptome profiling of log-phase cultures by microarray analysis. Genes listed were differentially expressed with a fold change ≥ 2.0 and P<0.05 in in reduced (250 μM) Mg2+ media, compared to high (2000 μM) Mg2+ media, in the indicated strain. Fold change values are averages of three independent experiments, P<0.05. (PDF) [file ppat.1004645.s009.pdf]

Strain	Rv #	Gene	FC	Description	Process
wt	Rv1806	*PE20*	3.6	PE family protein	
wt	Rv0166	*fadD5*	-2.3	Fatty-acid-CoA ligase	Metabolism
perM::tn	Rv1806	*PE20*	8.4	PE family protein	
perM::tn	Rv1807	*PPE31*	7.8	PPE family protein	
perM::tn	Rv1536	*ileS*	2.2	Isoleucyl-tRNA synthetase	Protein synthesis
perM::tn	Rv3810	*pirG*	2.0	Exported repetitive protein	Virulence
perM::tn	Rv1435c		2.0	Probable conserved proline, glycine, valine-rich secreted protein	
perM::tn	Rv0166	*fadD5*	-2.3	Fatty-acid-CoA ligase	Metabolism
