# Supplementary material for: Disruption of an M. tuberculosis Membrane Protein Causes a Magnesium-dependent Cell Division Defect and Failure to Persist in Mice
Source: PLoS Pathog. 2015 Feb 6;11(2):e1004645. doi: 10.1371/journal.ppat.1004645 (PMC4450064; doi:10.1371/journal.ppat.1004645)
Supplement: S3 Table — Nitrocefin activity is expressed as μg nitrocefin hydrolyzed min-1 (mg total protein)-1 ± SD. The differences between the strains were not statistically significant. Each assay was performed with triplicate cultures. (PDF) [file ppat.1004645.s010.pdf]

**Strain**	**Nitrocefin activity (nitrocefin hydrolysed min^{-1} mg total $protein^{-1}$)**
H37Rv	1.91 ± 0.245
perM::tn	1.54 ± 0.655
comp	2.01 ± 0.227
